# Effects of rosmarinic acid and doxorubicine on an ovarian adenocarsinoma cell line (OVCAR3) via the EGFR pathway

**DOI:** 10.1590/acb390524

**Published:** 2024-02-05

**Authors:** Umut Sarı, Fuat Zaman

**Affiliations:** 1 Umut Sarı Clinic – Department of Gynecology and Obstetrics – Istanbul, Turkey.; 2 Diyarlife Hospital – Department of Obstetrics and Gynecology – Diyarbakır, Turkey.

**Keywords:** Antioxidants, Epidermal Growth Factor, Ovary, Neoplasms, Cell Culture Techniques

## Abstract

**Purpose:**

We aimed to reveal the effects of rosmarinic acid (RA), which has come to the forefront with its antitumor and antioxidant properties in many studies recently in the ovarian adenocarcinoma cell line, on the epidermal growth factor receptor (EFGR) signaling pathway in the presence of doxorubicin (DOX).

**Methods:**

Ovarian adenocarcinoma cell line (OVCAR3) and human skin keratinocyte cell line human skin keratinocyte cell line (HaCaT) were used as control. (3-(4,5-dimethylthiazol-2-yl)-2,5-diphenyltetrazolium bromide (MTT) test was applied to determine the effect of RA and DOX on the proliferation of OVCAR3 and HaCaT cells. Bcl2 expression and epidermal growth factor receptor (EGFR) and western blot analysis were performed to determine the expression levels of the markers.

**Results:**

It was determined that RA (IC_50_ = 437.6 μM) and DOX (IC_50_ = 0.08 μM) have the ability to inhibit the proliferation of OVCAR3 cells and induce apoptosis in a 72-hour time and dose-dependent manner. Western blot showed that the expression level of Bcl-2 and EGFR in OVCAR3 cells was down-regulated by RA and DOX.

**Conclusions:**

Apoptosis in OVCAR3 cells can potentially be induced by RA via the EGFR pathway, and RA may be a potent agent for cancer therapy.

## Introduction

Ovarian cancer is the third most common gynecological malignancy worldwide. However, it has the highest mortality rate among cancers due to its asymptomatic course, late diagnosis, and recurrence^1^^–^ 3. When diagnosed, it is usually associated with omental involvement, diffuse malignant ascites, and intraperitoneal metastasis^2^^–^ 4. According to current data, ovarian cancer can develop resistance to traditional chemotherapeutics, which contributes to recurrence^5^. The specific etiology for ovarian cancer is still unknown, and the early molecular events underlying development are unknown as these cancers tend to occur in advanced stages.

Medicinal plants are important not only as therapeutic agents, but also for pharmacological research and drug development. Rosmarinic acid (RA) is a polyphenol found in many plants. It is especially found in high concentrations in oregano, rosemary, lemon balm, sage, and marigold. It is one of the aroma-giving components in these plants^6^. RA has very strong antioxidant activity. The antioxidant activity of RA is stronger than that of vitamin E. While reducing the risk of RA cancer and vascular occlusion, it prevents cell damage caused by free radicals^7^. The mutagenic or antimutagenic effects of various doses of RA against the antitumoral agent doxorubicin (DOX) in vivo in the peripheral blood cells of Swiss mice by the micronucleus (MN) method was investigated. No increase in MN frequency was observed in mice treated with three different doses of RA compared to the control group. A significant reduction in MN frequency was observed compared to animals given DOX in combination with RA. Although the mechanisms causing this protective effect are not fully known, it has been observed that RA exhibits strong antioxidant activity against DOX toxicity^8^.

Epidermal growth factor receptor (EGFR) is a 170 kDa glycoprotein that contains various signal transduction systems that affect cellular growth, differentiation, and proliferation. The EGFR is the first member of the receptor tyrosine kinase family, which consists of four structurally similar but functionally different receptors. All these transmembrane receptors contain intrinsic kinase activity activated by modified tyrosine residues. Binding of growth factors to the receptor stimulates kinase activity^9^. Preclinical studies have shown a strong correlation between increased EGFR expression and tumorigenesis. In some cases, even a change in EGFR levels alone may be sufficient to induce cancer development. Increased EGFR expression was also detected in breast, bladder, cervix, renal, ovarian, lung, and various squamous cell carcinomas^10^. Another mechanism that leads to an increase in EGFR expression is the activation of the receptor without ligand binding as a result of tumorogenic changes in EGFR activity through mutations. In-vivo studies have shown that spontaneous EGFR activation without ligand leads to tumor formation in transgenic animals^11^. In addition, the long-term survival rate for ovarian cancer has decreased, and the rate of recurrence has increased^12^.

In our study, a cell line from the most common epithelial subtype of ovarian cancer was used, the OVCAR-3 human ovarian adenocarcinoma cell line. Many preclinical studies have shown that there is a strong correlation between increased EGFR expression and tumorigenesis. It has been reported that, in some cases, changes in EGFR levels alone may be sufficient to induce cancer development. Unusual increases in EGFR expression have also been detected in breast, bladder, cervix, renal, ovary, lung, and various squamous cell carcinomas. For this reason, we think that it is important to determine the activities of newly researched agents on this pathway.

The anti-cancer effects of the application of DOX agent and RA antioxidant compound as single or in combinations were evaluated by performing cell cultures: cell viability test (MTT), quantitative real-time polymerase chain reaction (PCR), Western blot, matrigel invasion experiments. In order to reduce these problems, it is important to search for new drugs that are non-toxic and work in alternative/similar ways to these drugs, thus offering additional treatment options in ovarian cancer. One of these new agents is RA, a natural antioxidant.

## Methods

### Cells culture

In the study, ovarian carcinoma cell line NIH:OVCAR-3 (HTB-161™) and human skin keratinocyte cell line HaCat (RRID:CVCL_0038) were used as healthy cell line. OVCAR-3 cell line was cultured in RPMI 1,640 medium containing 10% fetal bovine serum (FBS), HaCaT cell line was cultured in Eagle’s miminal essential medium (DMEM) containing the same additives, and cells were grown in sterile incubators at 37°C and 5% CO_2_. All of the studies were started from the fifth passage of the cell lines, and the study was terminated at the 15th or 20th passage at most.

### MTT assay

The IC_50_ value of DOX and RA was determined by cell viability test. Then, after determining the maximum proliferation time, it was determined whether there was an inhibition related to the EGFR signaling pathway. The apoptotic pathway was also determined by protein analysis. It was then determined whether the antioxidant or antitumor effects of the combination of RA and DOX were activated in the OVCAR-3 cell line.

To determine the IC_50_ doses of DOX and RA, the OVCAR-3 and HaCaT cell line were seeded into 96-well culture dishes with automated multipipettes. At the end of one night (approximately 16 hours), DOX 0.5–50 μM and RA 10–1,000 μM were applied in nine different concentrations obtained by serial dilution, and the plates were incubated for 24, 48 and 72 hours. In MTT cell viability analysis, each chemotherapy agent and vehicle control groups consisted of six wells. MTT test was performed for cell survival (viability) analysis after incubation. For this, the yellow tetrazolium MTT (3-(4,5-dimethylthiazolyl-2)-2,5-diphenyltetrazolium bromide) test solution prepared at the dose of 5 mg/mL was pipetted into all wells at a rate of 20 μL/well. Then, the plates were left to incubate for 4 hours. After the incubation, the media in the wells were completely removed, and 200 μL of ultrapure DMSO (Merk, United States of America) was added to each well and left in the incubator in the dark for 2-4 hours. At the end of this period, the plates were read spectrophotometrically with a Multiskan GO microplate reader (Thermo Scientific, United States of America) at 492, 570 and 650 nm wavelengths. The value obtained from the control group applied to the vehicle mine was determined as the comparative viability rate based on 100% viability. IC_50_ values for each tumor cell line and chemotherapy agents in the control and experimental groups were calculated using the Statistical Package for the Social Sciences 20 program and probit analysis.

### Total RNA isolation and cDNA synthesis

In the study, OVCAR-3 cells were seeded in 25-cm^2^ culture flasks and incubated until the logarithmic phase was achieved. When the cells reached the logarithmic phase, vehicle control, DOX IC_50_ = 0.08 μM, and RA IC_50_ = 437.6 μM were administered singly and in combination. RNA was isolated from the samples 72 hours after the application. Purelink RNA mini kit (Thermo, United States of America) was used in the isolation stage, and the kit protocol was followed. The purity of the collected RNAs was determined by Optizen NanoQ microvolume spectrophotometer (Mecasys, South Korea), and all were equilibrated to 750 ng/10 μL with ultrapure water.

Complementary DNA synthesis was performed in order to ensure that the RNAs obtained after the synchronization process could be amplified by polymerase chain reaction (PCR). High-Capacity cDNA reverse transcription kit (Life Technologies, United States of America) was used at this stage.

### Quantitative real-time polymerase chain reaction

In the study, the expression levels of the EGFR gene responsible for EGFR/signaling pathways and the expression levels of the BCL-2 gene responsible for the apoptosis pathway in the control and administration groups of OVCAR-3 ovarian carcinoma cells were analyzed by proliferation quantitative real time PCR (qRT-PCR) method. The primers used to investigate the changes in the expression of these genes are given below in 5’-3’ order.

*EGFR: F: GCCAAGGCACGAGTAACAAGC, R: AGGGCAATGAGGACATAACC*

*BCL-2: F: ATGTGTGTGGAGAGCGTCAA, R: ACAGTTCCACAAAGGCATCC*

*Β-Aktin: F: CCTCTGAACCCTAAGGCCAAC, R: TGCCACAGGATTCCATACCC*

*GAPDH: F: CGGAGTCAACGGATTTGGTCGTAT, R:GCCTTCTCCATGGTGGTGAAGAC*

In gene expression studies, cDNAs obtained from RNAs isolated as described in the ‘Total RNA isolation’ section were used. These cDNAs were performed in qRT-PCR in accordance with the Power Sybeer Green qPCR MasterMix (thermo, United States of America) protocol. In the study, cDNAs were amplified using Applied Biosystems QuantStudio 5 Real-Time PCR device. Endogenous control glyceraldehyde 3-phosphate dehydrogenase (GAPDH) and β-actin mRNA expressions were used as calibration and correction factors with multiple control method.

### Western blot

In the study, OVCAR-3 cells were seeded into 75 cm^2^ culture flasks and incubated until the logarithmic phase was achieved. When the cells reached the logarithmic phase, vehicle control, DOX IC_50_ = 0.08 μM, and RA IC_50_ = 437.6 μM were administered singly and in combination. Protein isolation was done from the samples 72 hours after the application. In the study, Western Breze brand ready kits provided by Thermo Scientific firm were used, blotting and transfer to the membrane were carried out using the iBlot 2 (Life Technologies) system, ready-made membranes and kit, following the kit protocols. After blotting, the proteins were treated with Anti-EGFR (Phospho-Tyr1197) Antibody (ABM, CAT no: Y011228), Beta actin Antibody (Invitrogen, CAT no: MA1-140) specific primary antibodies, then antibodies were labeled with appropriate secondary and Micro Observed with the ChemiDoc (DNR Bio-Imaging Systems Ltd, United States of America) gel imaging system. Band intensities were calculated using GelQuant software.

### Statistical analysis

In the study, the difference between the live cell ratios determined by the MTT test and the averages of the expression values obtained by the 2-∆∆Ct method in qRT-PCR array studies were determined by one-way analysis of variance, and the groups in which the averages entered were determined by the Tukey HSD test. In the comparison of the two groups, depending on the homogeneity of the data, the dependent sample T test or the Mann-Whitney U test were used. Analyses were made with Statistical Package for the Social Sciences 20 (IBM, United States of America) and Graphpad programs, and p ≤ 0.05 was used.

## Results

**Figure 1:**
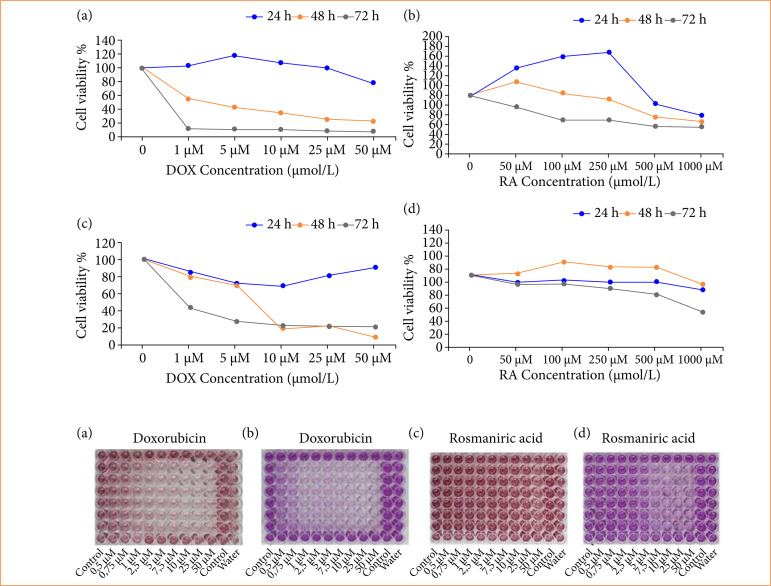
Full explanation of the figure. (a and b) Effect of DOX and RA at different times and at different concentrations on the survival rate of human ovarian carcinoma cells OVCAR3 and (c and d) human dermal keratinocyte cells HaCat (c ± S, n = 6).

RA: rosmarinic acid; DOX: doxorubicin; OVCAR3: ovarian adenocarcinoma cell line; HaCaT: human skin keratinocyte cell line. Source: elaborated by the authors.

The basal proliferation rate of OVCAR3 cells showed statistically significant differences compared to HaCat cells. The inhibitory effect of RA and DOX on proliferation of OVCAR3 cells increased in a time- and concentration-dependent manner (Figs. 1a and 1b). The IC_50_ of RA on OVCAR3 cells was not found at 24 h, but it was 980.3 μM at 48 h and 437.6 μM at 72 h (Table 1). On the other hand, in the HaCat cell line, the IC_50_ value of RA was not found, while the IC_50_ value of DOX was 5.32 and 1.23 μM at 48 and 72 hours, respectively (Table 1).

**Table 1:** IC_50_ of RA/DOX on OVCAR3 and HaCaT cells at different times.

Cell strains/RA, DOX	IC_50_ (μmol/L)		
	24 h	48 h	72 h
OVCAR3/RA	-	980,3	437,6
OVCAR3/DOX	-	2,12	0,08
HaCaT/RA	-	-	-
HaCat/DOX	-	5,32	1,23

RA: rosmarinic acid; DOX: doxorubicin; OVCAR3: ovarian adenocarcinoma cell line; HaCaT: human skin keratinocyte cell line. Source: elaborated by the authors.

Rosmarinic acid shows great therapeutic potential in many cancers, but its anti-cancer effect on the OCVAR3 cell line remains to be demonstrated. First, different concentrations of RA and DOX were used to treat human ovarian adenocarcinoma cells OVCAR3 for 24, 48 and 72 hours, and then cell viability was measured. As shown in Fig. 1a, cells that slightly increased at 24 hours of DOX administration showed a rapid concentration-dependent decrease at 48 and 72 hours. As shown in Fig. 1b, the activity of OVCAR3 cells decreased with increasing RA concentration. Cells that increased up to 250 μM/L at the 24th hour of RA application lost their viability rapidly depending on the concentration and time. Cell activity fell below 60% when the RA concentration reached 1,000 μM/L for all three different time periods. At 72 hours, cell activity decreased markedly for most concentrations. Therefore, IC_50_ RA and IC_50_ DOX incubation were selected for the analyses in this study.

**Figure 2:**
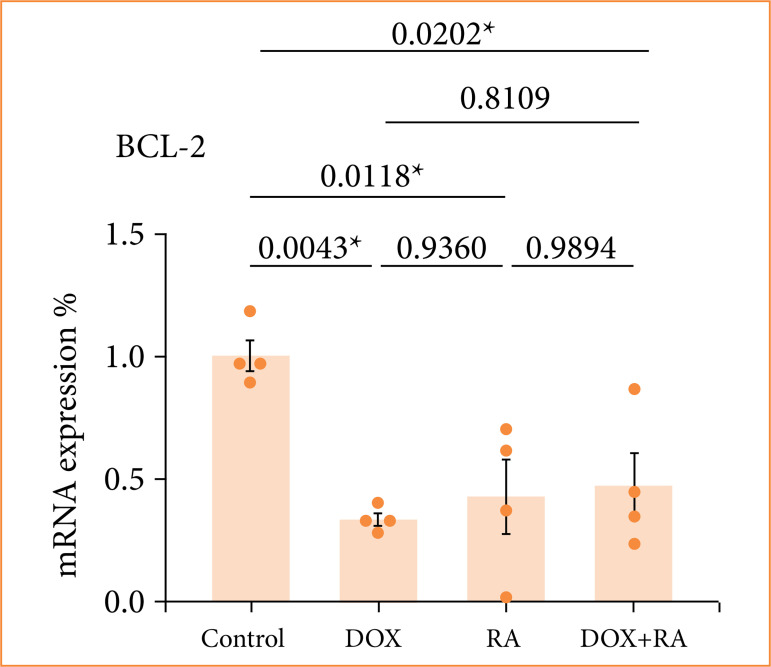
Relative fold increase values of Bcl-2 gene expressions in OVCAR-3 ovarian carcinoma cell lines.

OVCAR3: ovarian adenocarcinoma cell line; DOX: doxorubicin, IC_50_: 0.08 μM; RA: rosmarinic acid, IC_50_: 437.6 μM, 72 hours after single and combined drug administration. Source: elaborated by the authors.

To investigate the role of RA in the apoptosis of OVCAR3 cells, Bcl-2 gene expression was examined in our study. As shown in Fig. 2, normal OVCAR3 cells exhibited a low rate of apoptosis, while the rate of apoptotic cells increased with RA treatment. Also, the expression of Bcl-2 was decreased (Fig. 2b). Furthermore, cell viability was also significantly inhibited by DOX, and the presence of RA further enhanced this inhibitory effect, suggesting that RA may enhance the sensitivity of DOX in ovarian cancer cells.

**Figure 3:**
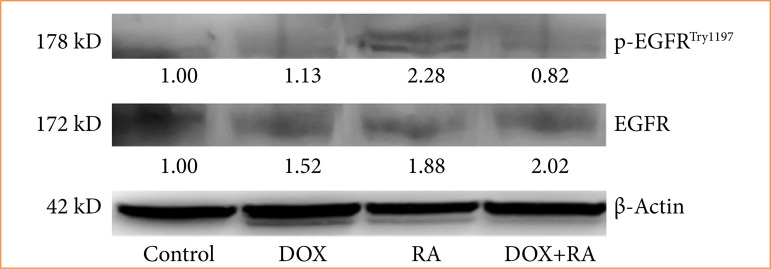
Within the scope of the study, vehicle control, DOX, RA, p_EGFR and EGFR protein levels were determined after 72 hours when OVCAR-3 was applied to ovarian carcinoma cells as a single or a combination.

OVCAR3: ovarian adenocarcinoma cell line; DOX: doxorubicin, IC_50_: 0.08 μM; RA: rosmarinic acid, IC_50_: 437.6 μM, 72 hours after single and combined drug administration; EGFR: epidermal growth factor receptor. Source: elaborated by the authors.

Next, we aimed to explore the possible mechanism underlying the inhibitory effect of RA on apoptosis and cell proliferation. As described in Fig. 3, protein expression of p-EGFR and EGFR was significantly inhibited by 72 h IC_50_ = 437.6 μM RA and IC_50_ = 0.08 μM DOX treatment.

## Discussion

In this study, the human ovarian carcinoma cell line OVCAR3 was used to investigate the effect and potential mechanism of RA on ovarian adenocarcinoma. Our results revealed that RA can inhibit cellular proliferation, migration, and invasion, as well as increase apoptosis and DOX sensitivity in OVCAR3 cells in vitro. RA also downregulates the expression level of p-EGFR, EGFR, and Blc-2 in OVCAR3 cells, suggesting that it has anticancer potential for the treatment of ovarian carcinoma, and the underlying mechanism may be related to inhibiting EGFR activation.

Ovarian cancer is the leading cause of gynecological cancer deaths in developed countries and often occurs at an advanced stage^13^. Current standard treatment of advanced ovarian cancer is cytoreductive surgery and platinum/taxane-based chemotherapy^13^^–^ 15. The response rate to treatment is about 80–90%, but most often relapse and develop resistance to chemotherapy^14^. Therefore, there is a need for alternative approaches for the diagnosis and treatment of ovarian cancer. Over the past three decades, a number of preclinical and clinical studies have been conducted to identify potential drug candidates (as mono or combined therapies) or to improve the therapeutic efficacy of existing chemotherapy regimens against ovarian cancer^16^.

Novel compounds derived from plants have recently gained importance as an alternative to traditional therapies due to their potent activity against carcinogenic cells with limited or negligible side effects. In particular, they focused on producing some promising cytotoxic drugs originating from natural compounds such as RA, alpha lipoic acid, ascorbic acid, and curcumin^17^^–^ 19. It is mainly aimed to reduce or even eliminate the side effects of existing chemotherapy. Antioxidant therapy can be defined as a treatment that prevents or reduces the side effects of free radicals. The effectiveness of exogenous antioxidants in protecting tissues from oxidative stress in vivo is variable and depends on the type of antioxidant, its biopharmaceutical properties, its concentration at the site of action, and the nature of oxidative stress^19^.

RA is a derivative of caffeic acid and 3,4-dihydroxyphenyllactic acid found largely in plants such as *Rosmarinus officinalis*, *Perilla frutescens* L. and *Salvia officinalis* L.^20^. Many studies have shown that RA has a wide range of pharmacological effects due to its antitumor, anti-inflammatory, antioxidant and antimicrobial properties^21^. RA plays an important role in inhibiting skin, pancreas, breast, lung, colorectal cancer, and leukemia^22^. It has been reported that RA inhibits multidrug resistance in human gastric cancer cells^23^. In other studies, it has been suggested that RA may increase the sensitivity of lung cancer and ovarian cancer cells and have synergistic effects with cisplatin^24^^,^ 25. Consistent with the results reported in these studies, the anticancer effect of RA on human ovarian adenocarcinoma cells was confirmed, promoted by RA-induced apoptosis in OVCAR3 cells and inhibition of proliferation and migration with DOX sensitivity.

It is well known that it results from overexpression of different tyrosine kinase receptors, including EGFR, and leads to the activation of oncogenic signaling pathways such as PI3K/AKT, ERK and mTOR^26^. RA plays an important anti-proliferative role, which is also associated with a decrease in EGFR expression. In addition to its inhibitory role, RA potentiates the activation of the Bax pathway by inhibiting Bcl-2, thereby increasing cancer cell apoptosis^18^.

Therefore, we wanted to examine the effect of RA on OVCAR-3 cells, especially on this pathway. According to our qRT-PCR results, among the gene groups in the p-EGFR, EGFR pathway, a decrease in EGFR expressions was detected in the group treated with RA alone and RA+DOX. Among these changes, the decrease in EGFR gene expression in RA, DOX, RA + DOX groups was found to be statistically significant (p < 0.05). Interestingly, the suppression of EGFR signaling pathway in the dual dose group (RA + DOX) revealed a curious effect of RA in ovarian cancer in the literature.

Unlike necrosis, apoptosis is the most common form of cellular death that can occur during various phases of embryonic development, tissue regeneration and regulation, and tumor regression^27^. Bcl-2 inhibits apoptosis, and Bcl-2 protein family has two types according to its function^28^. The first type is Bcl-XL, Bcl-W, Mcl-1 and A1, which are similar to Bcl-2 and inhibit apoptosis. The other type is those that trigger apoptosis such as BAX, Bcl-Xs, Bik/Nbk and Bid^29^. Our research showed that RA can inhibit the expression of the apoptosis factor Bcl-2. From this point of view, we thought that RA performs apoptosis through the mechanism of decrease in Bcl-2 gene expression.

When we evaluate all this literature review and our results, it seems that RA plays two opposing roles as a ROS scavenger. Depending on the concentration and redox modulation, it can act as an antioxidant or pro-oxidant. While protecting normal cells by eliminating ROS at low doses, at high doses it induces apoptosis and cytotoxicity in cancer cells^30^. It has been also shown that RA has much stronger anti-cancer effects when combined with chemotherapeutic agents or DOX. In our study, RA also affected many steps on proliferation, migration, apoptosis, cell cycle, EGFR pathway gene expressions, Bcl-2 protein levels and apoptosis mechanism. It also increased the efficiency of the RA + DOX duo in OVCAR-3 cells, as we expected.

## Conclusion

It is important to increase the sensitivity of existing chemotherapeutic drugs due to the increasing incidence of cancer and the difficulties encountered in treatment. Alternative methods that can be applied at lower doses without sacrificing the effectiveness of drugs are being investigated. It is of great importance to be able to explain apoptosis and cell cycle-related complex mechanisms in terms of new potential agents that have few side effects and can increase their potency together with existing drugs. Therefore, an investigated compound, RA, has been shown to exhibit anti-tumor activities in various cancer models by affecting multiple steps in most of the signaling pathways related to proliferation, invasion, migration, EMT and apoptosis.

## Data Availability

All data sets were generated or analyzed in the current study.
